# A dose‐gradient analysis tool for IMRT QA

**DOI:** 10.1120/jacmp.v6i2.2006

**Published:** 2005-05-21

**Authors:** Jean M. Moran, Jeffrey Radawski, Benedick A. Fraass

**Affiliations:** ^1^ Department of Radiation Oncology University of Michigan Medical Center Ann Arbor Michigan 48109‐0010 U.S.A.

**Keywords:** dosimetric analysis, gradient calculation, gradient compensation, IMRT, QA

## Abstract

The use of intensity‐modulated radiation therapy (IMRT) has led to an increase in the number of complex fields that require measurement and comparison to calculated dose distributions in 2D. Current dose evaluation techniques, including isodose line comparisons, displays of the dose difference between calculated and measured distributions, and distance‐to‐agreement (DTA) comparisons, are useful for display of differences between two different dose distributions but are often of limited value for the assessment of the discrepancies in terms of significance and/or cause. In this paper, we present a new gradient compensation method for the evaluation of local dosimetric differences as a function of the dose gradient at each point in the dose distribution. To apply the method, the user specifies a distance parameter (typically 1 mm), which is the geometric tolerance the user is prepared to accept for the dose comparison. The expected geometric uncertainties in the comparison process can include finite calculation and measurement grids, small misalignments of measured and calculated results, and volume‐averaging effects in the measurement detector. Since these uncertainties can obscure the interpretation of any of the analysis tools described above, removing dose differences related to the tolerable geometric uncertainty helps the gradient compensation method highlight algorithm and delivery‐related differences. The remaining dose differences not explained by the geometric tolerance can then be evaluated graphically (dose difference display) or analytically (dose difference dose‐volume histograms) over the entire comparison region.

PACS number: 87.53.Xd

## I. INTRODUCTION

The clinical implementation of intensity‐modulated radiation therapy (IMRT) has required patient‐specific measurements prior to treatment at many centers. Recent guidance documents published by the IMRT Collaborative Working Group,^(1)^ AAPM,^(2)^ and a joint AAPM and ASTRO working group^(3)^ discuss approaches used to verify patient IMRT treatment fields prior to delivery. Typical measurement‐based procedures for dosimetric verification include two steps: (1) point dose measurement with an ion chamber in a phantom for a delivery including all treatment fields at the correct gantry and collimator angles for a high dose point in a shallow gradient region of the dose distribution, and (2) 2D planar dose measurements at depth in a simple phantom at a standard gantry angle.^(2)^

While guidelines from the AAPM and ASTRO clearly state recommendations for dosimetric agreement for point dose measurements, it is more difficult to specify agreement criteria for planar dose comparisons. The steep dose gradients common in IMRT fields make it difficult to interpret dose differences using standard comparison tools (isodose overlays, 2D dose difference displays, and dose area or volume histograms).^(4^ ^–^ ^6)^ For example, Fig. 1 shows (a) an intensity pattern and (b) the corresponding isodose line comparison between calculation and measurement for that field. Most of the isodose lines show reasonable agreement over most of the field (e.g., within 2 mm); however, only a limited number of isodose lines have been displayed (due to the complexity of the dose distribution), thereby making it difficult to fully evaluate the agreement between calculation and measurement in all parts of the dose distribution. The isodose line display also contains little information about how large discrepancies are when the lines are not coincident. Another way to view the differences is to use a dose difference display ((Fig. 1c). This method is sensitive to small dose discrepancies but may show large differences due to the effects of the dose calculation or measurement grids, due to volume averaging in the detector or due to small (<1 mm) geometric misalignments in steep gradient regions. As a result, it is often difficult to determine whether a specific dose difference is significant. Distance‐to‐agreement (DTA) and the gamma evaluation (combination of DTA and percent dose difference) are often used with a single tolerance value to try to summarize the overall quality of the agreement between distributions.^(6, 7)^

**Figure 1(a):**
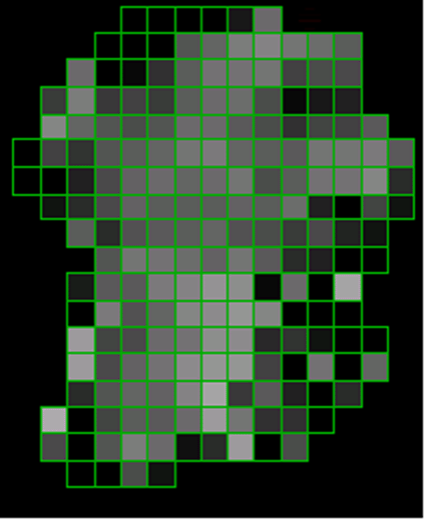
Example fluence map.

In this work, a new dose evaluation tool is described to evaluate local dosimetric differences based on the dose gradient at each point in the field. This technique was developed specifically for IMRT field evaluation because the significance of differences may depend on the local gradients. Known geometric effects, such as finite size of measurement and calculation grids and small spatial misalignments, may dominate the quantitative analysis of any of these comparison techniques and therefore bias or confuse the assessment of calculation and measurement dose differences. When the effects of the local dose gradient are taken into account, these known effects can be removed from the dose distribution comparison, making evaluation of the remaining differences more obvious and straightforward.

## II. Methods

All measurements were performed with a 6 MV beam on a Varian 21 EX LINAC equipped with a Millennium multileaf collimator (MLC). The Millennium MLC includes 60 leaf pairs, where the middle 40 leaves are 0.5 cm wide, and the remaining leaves are 1 cm wide. For each measurement, a set of films was exposed to known doses at 10 cm depth in a $30\times 30{\;\text{cm}}^{2}$ solid water phantom (RMI, Madison, WI) to create a characteristic curve to convert optical density to dose. Film measurements were made at 5 cm and 10 cm depths for a range of head and neck IMRT fields at a gantry angle of 0° and 90 cm source‐to‐detector distance using XV‐2 film (Kodak, Rochester, NY). For each film, four pinholes were placed outside of the beam along the central axis crosshairs (using a jig) for alignment of the dosimetry film to the calculated distribution within the treatment planning system. Films were scanned using a laser film digitizer (Lumisys Lumiscan LS75, now Kodak, Rochester, NY). Film measurements were converted to dose using the characteristic curve described above. Films were imported in our in‐house 3D treatment‐planning system, UMPlan,^(8)^ and aligned using the pinholes described above. All dosimetric analysis was performed in UMPlan. IMRT fields were generated using our in‐house inverse planning/optimization system, UMOpt.^(9,^, ^10)^ IMRT fluence maps ($1\times 1{\;\text{cm}}^{2}$ beamlets) from the inverse‐planning system were delivered using step‐and‐shoot or segmental MLC (SMLC) delivery techniques, based on an in‐house MLC sequencer^(11)^ using a SMLC algorithm based on Bortfeld's method.^(12)^ All dose calculations were performed using a multiple component convolution/superposition algorithm based on the work of Mackie et al.^(13)^ The dose calculation grid size was 2 mm for the examples in this work.

Calculations and measurements were compared using the following tools: extracted isodose lines, dose‐difference displays, dose‐area histograms, and gradient compensation. The measurements and calculations were not renormalized with respect to each other. The comparisons are displayed in centigrays.

## III. DOSE‐GRADIENT AND GRADIENT COMPENSATION

Two new features have been implemented in the planning system for this work: (1) generation of the dose‐gradient distribution (in 2D or 3D) and (2) dose‐gradient compensation (for analysis of dose distribution comparisons). The gradient used in this case is the generalized gradient, defined as the sum of the squares of all the local gradients around each grid point. This generalized dose gradient can be calculated, saved, displayed, and histogrammed for 2D or 3D dose grids. To calculate the dose gradient for a calculated 2D or 3D grid, the dose difference is calculated between each dose grid point and its nearest neighbors on the calculation grid. The gradient at each point is then calculated with Eq. (1):

(1) $$G_{i}=Gradient=\sqrt{\sum {\left(\frac{\Delta d_{ij}}{\Delta x_{ij}}\right)}^{2}},$$

where $G_{i}$ is the generalized gradient at grid point *i*, $\Delta d_{ij}$ is the difference in dose between the grid point *i* and each of its nearest neighbors *j*, and $\Delta x_{ij}$ is the distance between the grid point *i* and each of the nearest neighbor points *j* used in the calculation. Four neighboring points are used for the gradient calculation in 2D and 6 points for the calculation in 3D. The generalized gradient is a positive scalar quantity and simply describes the magnitude of the variations, not the directional behavior of the local gradients. In 2D planar analysis, the gradient is calculated only over the displayed dimensions, while in 3D mode, the gradient is determined using all three dimensions. The gradient is given in dose units per millimeter, where the dose units (gray, centigray, percent) depend on the dose units or normalization of the original dose distribution.

The gradient compensation method modifies the dose difference distribution (created by subtracting two dose distributions, typically a calculation and a measurement) to remove differences that may be caused by geometrical mismatches of a given size (typically in our work, we evaluate the effect of choosing the distance parameter $d_{\text{gc}}$ to be 1 mm) that can be due to small misalignment of film or dose calculation grids. There are many geometrical discrepancies between measured and calculated dose distributions, which may lead to rather large differences in the dose difference distribution, especially in large gradient regions of the dose distribution, and the gradient compensation over a distance of interest allows the analysis of the dose difference with those differences removed. Gradient compensation is performed with the following steps:

1. The user creates the dose difference distribution between calculation and data, including performing any registration that is necessary to optimally align the two dose distributions with respect to the beam (since the gradient compensation tool is not useful for registration of the two distributions).
2. Calculate the gradient map for the calculated dose distribution.
3. Choose a distance parameter $d_{\text{gc}}$ (typically 1 mm), which is the size of the geometric uncertainty (dose differences caused by a geometric shift of this distance will be removed from the dose‐difference distribution by the gradient compensation).
4. Perform the gradient compensation.

Step 2 is the gradient map calculation described above, and step 4 simply calculates $G_{i}d_{gc}$ and decreases the dose difference at that point by the product. The dose difference at each grid point is thus reduced by the difference that could be caused by a geometrical effect with size $d_{\text{gc}}$. These effects could be remaining geometrical alignment differences, dose grid size effects, measurement resolution issues, etc. This basically removes the part of the dose difference that may be explainable by a geometrical error of size $d_{\text{gc}}$.

## IV. RESULTS

To demonstrate how the gradient calculation and resulting gradient compensation method works, a simple open field was calculated and normalized to 100 cGy ((Fig. 2a). Then the gradient is calculated for each point across the field ((Fig. 2b). Note that the gradients in the center and outside regions of the field are 0 because the dose is homogeneous in those regions. As expected, the dose gradient increases near the field edge and then decreases outside of it. For example, near the 50 cGy line the dose gradient is approximately 12 cGy/mm. To illustrate how the gradient compensation method works, the original dose distribution ((Fig. 2a) was shifted by 2 mm laterally and then subtracted from the original dose calculation. This results in a dose difference distribution ((Fig. 2c) with hot and cold spots up to 30 cGy near both field edges. Then the gradient compensation method is applied ((Fig. 2d) with $d_{\text{gc}}=1\;\text{mm}$. As expected, the dose differences are reduced (in this case to ±16 cGy). When gradient compensation is applied with $d_{\text{gc}}=2\;\text{mm}$ (the size of the actual shift between the two distributions), there is no longer a difference between the two distributions ((Fig. 2e), demonstrating that all the differences between the two distributions are explained by a geometric error of 2 mm, as it should for this simple example.

**Figure 1(b):**
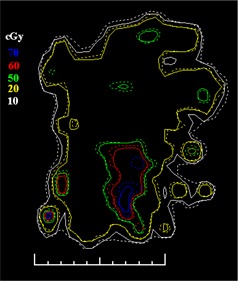
Isodose lines: calculated (solid); measured (dashed). The circled regions show regions of disagreement greater than 2 mm.

**Figure 2(b):**
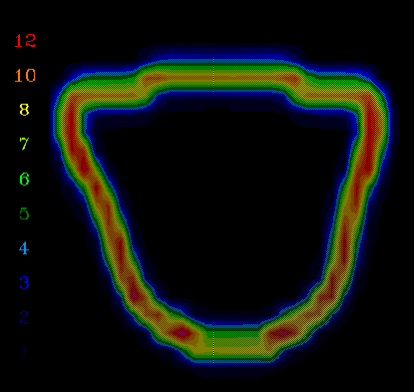
Gradient calculation. The gradient is highest at the field edge.

**Figure 2(c):**
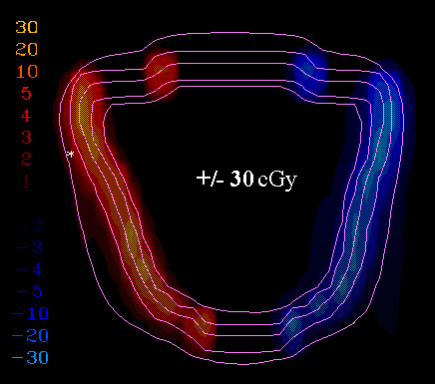
Display of difference between dose calculation shown in (a) and the same distribution shifted by 2 mm laterally.

**Figure 2(d):**
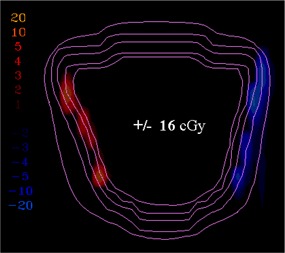
Dose difference display when 1 mm gradient compensation is applied.

**Figure 2(e):**
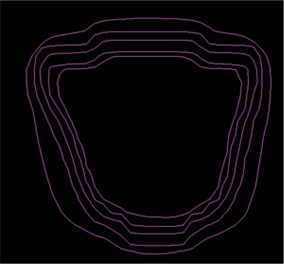
Dose difference display when 2 mm gradient compensation is applied.

In clinical practice, the gradient compensation method has been applied to evaluation of the dosimetric differences for IMRT fields as part of commissioning dose‐calculation algorithms and leaf sequencing algorithms and for individual patient pretreatment quality assurance (QA). Application of the gradient compensation method for an IMRT field is shown in Fig. 3 for the intensity pattern from (Fig. 1a). (Figure 3a) shows the calculated dose distribution for the field, at a depth of 10 cm in a flat phantom, and (Fig. 3b) shows the calculated gradient map. The dose difference display (calculation – measurement) is shown in (Fig. 3c). Notice that there are several regions where the calculations are lower than the measurements and vice versa. Because the leaf sequencer does not account for MLC tongue‐and‐groove effect and other dose calculation model limitations, there are differences in the lower dose regions of the field. When a 1 mm gradient compensation is applied ((Fig. 3d), many of the regions of large dose difference are seen to be potentially explained by geometrical errors of less than 1 mm. Nevertheless, other differences including those due to tongue‐and‐groove effect (4 cGy) are still seen (circled on the figure), since they cannot be explained by simple geometrical misalignments, even though the dose differences are relatively small. The dose difference that remains after 1 mm gradient compensation is evaluated using a dose area histogram ((Fig. 3e), limited (in this case) to the part of the field that receives 10 cGy and higher.

**Figure 1(c):**
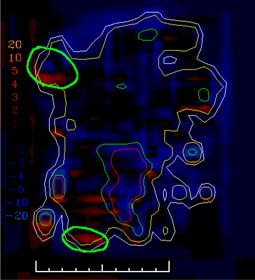
Dose difference display: calculations – measurement (cGy).

**Figure 3(a):**
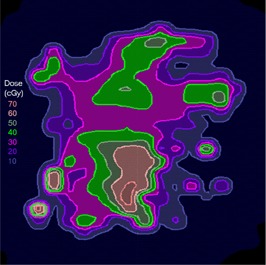
Dose distribution.

**Figure 3(b):**
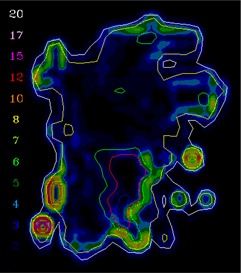
Gradient calculation.

**Figure 3(c):**
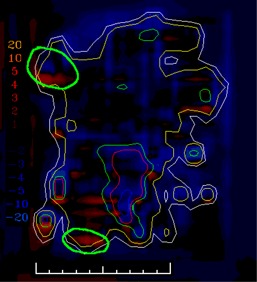
Dose difference display (calculation – measurement). Region of tongue‐and‐groove discrepancy circled.

**Figure 3(d):**
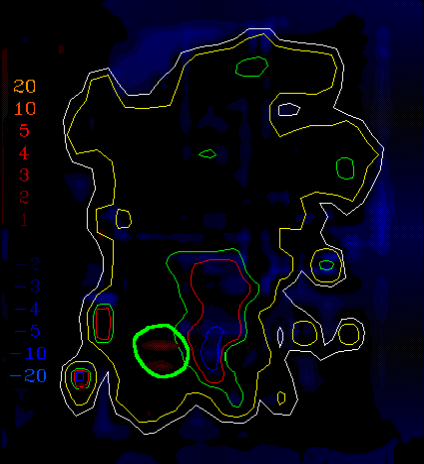
One millimeter gradient compensation applied to dose difference display. Some of tongue‐and‐groove effect is still seen.

**Figure 3(e):**
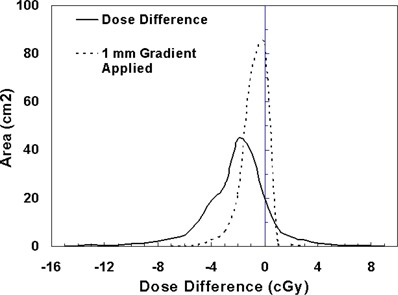
Dose area histograms for standard dose difference display (solid line) and when 1 mm gradient compensation is applied (dashed line).

(Figure 4a) depicts the fluence map for another head and neck IMRT field example, illustrating the evaluation of sequencing for this split field (the limitations of the MLC require this field to be split into two halves). This field contains high doses (up to 70 cGy) and steep dose gradients, as shown in the dose calculation ((Fig. 4b). The dose difference between the calculations and measurements is displayed in (Fig. 4c) with a dose colorwash. In this example, there are more regions where the measured dose is higher than the calculated dose, but it is unclear from the dose difference display if the discrepancies are significant. In (Fig. 4d), the gradient map for the calculation shows regions of steep dose gradients with a maximum of 20 cGy/mm.

**Figure 2(a):**
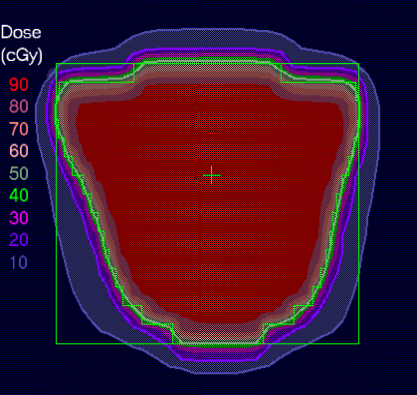
Dose colorwash display of a single prostate field.

When the 1 mm gradient compensation is applied ((Fig. 4e), gradient‐based uncertainties are removed, leaving some remaining dose differences that appear to be due to discrepancies between the modeling, sequencing, and measurement of transmission, all issues that will need to be included directly into the optimization process in order to further improve the agreement between calculation and data. This example illustrates the ability of the compensation method to make possible more detailed analysis of the comparison results, hopefully leading to improved algorithms and IMRT delivery.

## V. DISCUSSION

Assessing whether the agreement between dose calculation and measurement is clinically acceptable for IMRT has been a challenge in clinical practice. The commonly used dose analysis tools are typically not adequate for a full analysis of the IMRT dose distribution comparisons. Isodose line comparisons only show the agreement at the displayed isodose levels and give no information in regions between comparable lines. Dose difference displays show all the dose differences but are very sensitive to high dose difference values in the high gradient regions, caused by small geometric effects. Distance‐to‐agreement is useful in high gradient regions to show possible geometric effects but is much less helpful in low gradient regions. Quantitative evaluation of each of these analysis methods is difficult, for both analysis during commissioning and routine patient QA use.

The gamma dose distribution comparison method is another evaluation tool that has been applied for comparison of measured and calculated IMRT distributions.^(6,^, ^7)^ For this technique, the user chooses acceptance criteria for both the dose difference and the DTA. The method then generates a map of regions of the measured plane that fail both of the chosen criteria. For 2D evaluations, the magnitude of the overall disagreement is displayed in 2D. This technique allows the user to judge differences in dose and DTA simultaneously, for a single choice of dose and DTA criteria. This technique has been applied to IMRT field evaluation using pass/fail criteria for the evaluation of measured portal dose images and calculations.^(14)^ An advantage of the gamma technique is that it provides a quick evaluation of regions of either dosimetric or spatial disagreement. However, it can be difficult to evaluate in detail other disagreements in dose or geometry. Also, the method is sensitive to noise in the reference or evaluated frames and to the effect of calculation and measurement grid sizes, often requiring use of clinical evaluation criteria of 3% to 5% and 2 mm to 3 mm.^(7)^

The gradient compensation method uses a different approach to take into account spatial (including grid effects) and dosimetric differences. First, only a given, small spatial disagreement (typically 1 mm) is considered, to compensate for the effect of the calculation and measurements grids (a distance value of one‐half of the calculation grid can be used) and the potential for small misalignment when registering the measurement. Then local dosimetric disagreements that remain after calculation of the gradient over the distance parameter of interest are displayed and evaluated. Dose differences that remain in the dose difference display after local gradient compensation should be explored in further detail since the disagreements may be due to undetected shortcomings in the calculation algorithm, sequencing, measurement (technique, data analysis, or detector used), or delivery methods.

The example comparisons shown in Figs. 3 and 4 demonstrate the usefulness of the method in highlighting differences due to algorithm deficiencies or delivery effects. For example, Fig. 3 shows the significance of using (1) a leaf sequencing algorithm that does not account for MLC tongue‐and‐groove effect design and (2) a dose calculation algorithm that does not completely model all aspects of the MLC and treatment head. (Figures 3d)and (4e) also demonstrate that the calculation model does not adequately account for the transmission through the MLC for the IMRT delivery. Other sources of discrepancies between calculations and measurements may also be caused by the sequencing and delivery of the fields. The gradient compensation tool is useful in distinguishing these types of differences from more straightforward geometric effects.

**Figure 4(a):**
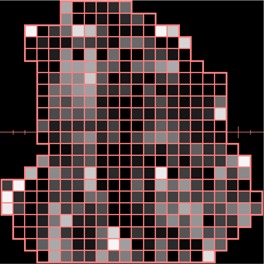
Example fluence map.

**Figure 4(b):**
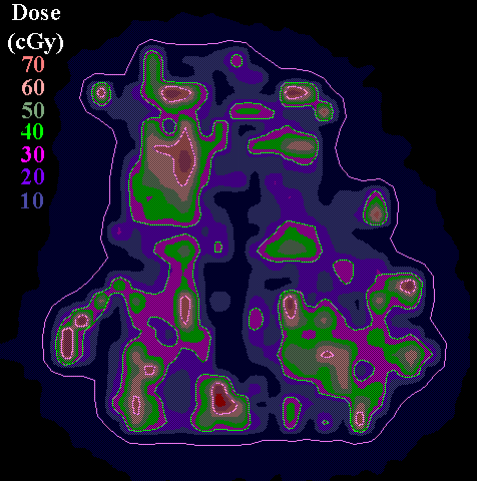
Dose distribution.

**Figure 4(c):**
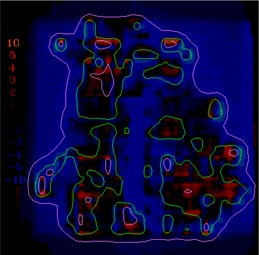
Dose difference display.

**Figure 4(d):**
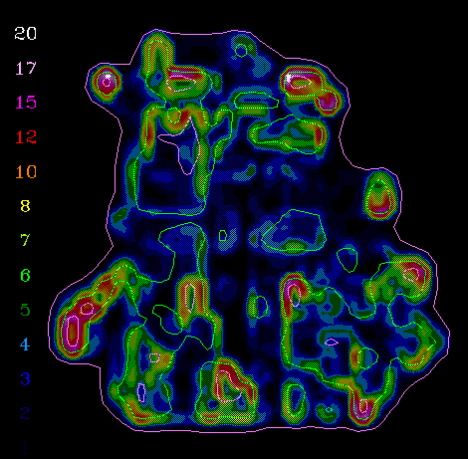
Gradient calculation.

**Figure 4(e):**
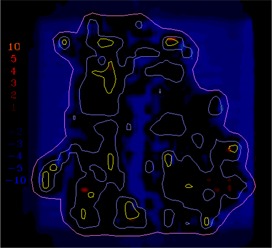
Dose difference when 1 mm gradient compensation applied.

The gradient compensation method can also be used quantitatively. For example, (Fig. 3e) shows a dose difference histogram over the region of interest with and without gradient compensation. The dose difference histogram for the gradient compensated evaluation is more useful than that done for the dose difference alone because the small (<1 mm) geometric and calculation grid errors have been removed. In the context of algorithm commissioning (dose calculation and leaf sequencing) and for routine pretreatment QA, the gradient compensation method is an efficient method for evaluating calculations and measurements.

The gradient compensation method is designed to be used in conjunction with other analysis methods such as dose difference displays. To ensure the method does not mask errors in positioning (greater than the reproducibility of positioning), it is important that measurements are aligned in the evaluation software independent of the dose calculation geometry and that other geometric errors in delivery or positioning are handled elsewhere in the QA process. As mentioned earlier, this can be accomplished with fiducial marks on film, such as pinholes for alignment. When reviewing the results of the gradient compensation, it is helpful to have an understanding of the limitations of the dose calculation algorithm and any approximations made in beam modeling. The value chosen for the distance parameter (typically 1 mm in our clinic) should represent spatial inaccuracies of the film measurement, alignment of film in the analysis software, and the discrepancies between the dose interpolation grids used for the calculations and the measurements.

## VI. CONCLUSIONS

In this work, we have described a new tool for dosimetric evaluation of IMRT measurements and calculations: gradient compensation. The dose‐gradient compensation method is a flexible and efficient tool for dosimetric analysis of dose calculation and leaf sequencing algorithms. The method should be used in conjunction with other dosimetric displays such as dose difference and isodose overlay displays. The technique is very useful in separating dosimetric differences from simple geometric differences due to misalignment of film or the size of the dose calculation grid, making quantitative analysis and evaluation of dose distribution differences easier to perform. This method has been very useful for both clinical commissioning and routine patient IMRT QA testing.

## ACKNOWLEDGMENTS

This work was supported in part by NIH grant P01‐CA59827.
